# Global budget payment system helps to reduce outpatient medical expenditure of hypertension in China

**DOI:** 10.1186/s40064-016-3565-7

**Published:** 2016-10-26

**Authors:** Yi Huang, Yan Liu, Xingyi Yang, Jing Li, Pengqian Fang

**Affiliations:** School of Medicine and Health Management, Tongji Medical College, Huazhong University of Science and Technology, Wuhan, Hubei People’s Republic of China

**Keywords:** China, Global budget, Fee for service, Medical expenditure, Hypertension, Health care reform

## Abstract

**Background:**

As healthcare spending continues to increase, medical insurance is now under great pressure of growing economic burden. To control the excessive growth of medical expenditure, change of medical payment system was clearly put forward in China’s new healthcare reform. With this end, Tianjin, a large city in North China, is now exploring to replace traditional fee-for-service (FFS) with global budget payment system (GBPS), and actual effects of GBPS needs to be assessed.

**Methods:**

Data of this study is from the 2013 National Health Services Utilization Survey among patients of Urban Basic Medical Insurance in China, containing 102,492 outpatient visits of 21,925 hypertensive patients to Tianjin’s primary hospitals in 2013. *t* test was used to compare the difference between continuous variables. A linear regression analysis was also done to identify possible risk factors of medical expenditure.

**Results:**

On the basis of expenditure per capital, GBPS, compared with FFS, has significantly reduced total medical expense (CNY 640.28 vs. CNY 700.64, *p* < 0.001), medical insurance (MI) fund expense (CNY 491.87 vs. CNY 532.37, *p* < 0.001) and out-of-pocket (OOP) expense (CNY 148.42 vs. CNY 168.27, *p* < 0.001). Results of generalized linear regression also show that younger people, female and GBPS independently predict less total medical expense, MI fund expense and OOP expense.

**Conclusions:**

Compared with FFS, GBPS can help reduce total medical expense, MI fund expense and OOP expense significantly. This study offers evidence for wider implementation of GBPS in China.

## Background

Nowadays, the rising medical costs have become a worldwide issue, and China is under an even greater financial burden. Total health expenditure per capital in China was reported to be USD 43 in 2000 and USD 274 in 2011, with an increasing rate of 537.21% over the 11 years. However, the increasing rates over the same period were only 137.41% for Western Pacific regions and 264.29% for upper middle-income countries (WHO 2014a). The fast rising medical cost adds great pressure to China’s insurance system. From 2000 to 2013, the annual revenue of Urban Employee’s Basic Medical Insurance (UEBMI) increased from CNY 17 billion to CNY 706.2 billion, with an average increasing rate of 33.20% per year. While during the same period, the annual expenditure of UEBMI increased from CNY 12.5 billion to CNY 58.30 billion, with an average increasing rate of 34.39%. The higher rising rate of expenditure compared with revenue confers risk for deficit of China’s medical insurance system (Fang 2014). Thus, containing the increasing trend of medical expenditure has become an urgent affair for China.

In terms of methods of reimbursement, health care providers in China have traditionally been paid on a fee-for-service (FFS) basis. Under this FFS payment system, medical suppliers have the incentives to increase the volume and intensity of services and to choose treatments with a greater profit margin, such as offering over-priced services and prolonging hospitalization (Schroeder and Frist 2013). With this end, change of medical insurance payment system was clearly proposed in China’s new medical reform plan released in 2009 (http://www.gov.cn/zwgk/2012-03/21/content_2096671.htm).

Global budget payment system (GBPS) offers a prospective reimbursement to a health care provider and the total expected spending was determined ahead of a budget year mainly based on the provider’s patient population. Many believed that GBPS, with its inherent financial risk for health care providers to assume the excessive expenses, offers stronger incentives to contain the growth of medical expenditure, and policy makers are now exploring to expand the use of GBPS for replacing traditional FFS (Markovich 2012). Initial results from America show the cost control effects of GBPS to be promising (Song et al. 2014).

Tianjin is an old industrial city in North China, with a large numbers of retirees and heavy burden of medical expenditure. There has been financial deficit for the medical insurance system of Tianjin since 2010 (Zhang and Yuan 2015). Human Resources and Social Security Bureau (HRSSB) of Tianjin began to explore effects of GBPS in 2004. Based on the initial experience gained from 5 pilot hospitals, HRSSB of Tianjin extended GBPS to 40 more hospitals of secondary or tertiary level in 2006 (Qiu 2009). Since 2011, The Ministry of Human Resources and Social Security of China issued advice on further pushing forward the reform of medical insurance payment method (http://www.mohrss.gov.cn/yiliaobxs/YILIAOBXSzhengcewenjian/201105/t20110531_83732.htm), and the range of hospitals joining GBPS has then been extended to parts of primary hospitals in Tianjin.

The practical regulations of GBPS in Tianjin can be summarized as follows. HRSSB of Tianjin negotiates with hospitals at the end of a year, and signs contracts to determine the total medical budget of the next year. The principles of building contracts include balancing revenue and expenditure, limiting total budget, sharing risk between health insurance fund and service providers and reserving surplus for providers’ self-use. The budget for each hospital depends on its volume and quality of service. So long as medical insurance fund is without deficit, there can be a 5–10% increase for the budget of next year. HRSSB appropriates 94% of the monthly budget to the hospital ahead of each month. The rest 6% is a reservation for the year-end assessment (Duan and Ren 2011).

Health care providers are expected to assume risk for expenditure exceeding the predetermined global budget, and risk sharing rules are as follows: (1) health care providers receive no reimbursement for expenditures exceeding annual budget that violate norms set by HRSSB for critical control indexes; (2) for the exceeding expenditure shared between HRSSB and health care providers, distribution ratio is 70:30 for the part of exceeding cost below 10% of the predetermined budget, 30:70 for the part above 10% and below 20%. Health care providers receive no reimbursement for the part exceeding 20% (Duan and Ren 2011). These rules are intended to raise the initiative of health care providers to curtail medical expenditure and increase the efficiency of medical insurance budgets.

Hypertension is one of the major risk factors for global mortality and is estimated to cause 9.4 million deaths per year according to the WHO Global Status Report on Non-communicable Diseases in 2014 (WHO 2014b). The prevalence of hypertension in the adult population varies from 5.2 to 70.7% worldwide, and it is estimated that more than 1.5 billion individuals can be currently diagnosed with hypertension (Danaei et al. 2011). So long as the diagnosis is definite, it is recommended for patients to take anti-hypertensive drugs and control blood pressure for the prevention of serious cardiovascular events. Since primary hospitals are easy to access and can meet most hypertensive patients’ demands of diagnosis, treatment and recheck, they play a pivotal role in the treating process of hypertension and has been laid upon a major role in China’s recently proposed grading treatment (http://www.nhfpc.gov.cn/yzygj/s3593g/201512/073b50bd7d2b4454872126f2bc830410.shtml).

As a chronic disease, hypertension is not only the leading risk factor for cardiovascular disease and premature death, but also a heavy economic burden for patients and medical insurance fund (Druss et al. 2001). The prevalence of hypertension is high in China and has been increasing in recent years, which adds to the ever increasing medical expenditure. In 2003, the prevalence of self-reported hypertension was 2.62% (5.47% in urban areas and 1.64% in rural areas), and average direct medical spending per visit was CNY94.1 based on the 3rd National Health Services Survey conducted among 57,000 families in China (http://www.nhfpc.gov.cn/mohwsbwstjxxzx/s8211/201009/49162.shtml). In 2008, the prevalence of self-reported hypertension increased to 5.49% (10.08% in urban areas and 3.85% in rural areas), and average direct medical spending per visit increased to CNY156.3, based on the 4th national health services survey conducted among 56,400 families (http://www.nhfpc.gov.cn/mohwsbwstjxxzx/s8211/201009/49165.shtml).

Theoretically, GBPS is predicted to be an effective way to contain increasing medical expenditure, however, relatively few studies have explored its actual cost control effect in mainland China, especially when China’s huge financial burden and urgent need to devise cost containment strategies are concerned. Gao et al. has shown that GBPS helps to reduce inpatient OOP and decrease the length of hospital stay, while contributing no significant benefit to total inpatient medical expenditure (Gao et al. 2014). Although studies from other regions have generally shown that GBPS can be beneficial for cost containment (Markovich 2012; Song et al. 2014; Chang et al. 2011), we still need more evidence before it can be widely implemented. In this paper, we drew data from hypertensive outpatient service of Tianjin in 2013 and try to explore the effect of GBPS on medical expenditure compared with FFS.

## Methods

### Data source

We used data from the 2013 National Health Services Utilization Survey (NHSUS) among patients with Urban Basic Medical Insurance, which is a nationwide representative survey of health care providers for expenditures of patients based on the classification of disease. This survey was conducted in 60 different areas among 29 provinces, and finally obtained 396 thousand effective piece of information, accounting for 0.75% of total population joining Urban Basic Medical Insurance in China. NHSUS among patients with Urban Basic Medical Insurance was sponsored by China Health Insurance Research Association, and has been carried annually since 2008.

The data of this research was a random selection of total pool of survey results in Tianjin, containing 102,492 visits of 21,925 hypertensive patients to the outpatient departments of 479 primary hospitals. One selection of patient covers all his visits this year, and the HRSSB has set a fixed primary hospital for his outpatient reimbursement. In the survey, hypertension was defined as those with a systolic blood pressure not less than 140 mmHg, or a diastolic blood pressure not less than 90 mmHg, or a self-reported medication for hypertension. Data from a visit covers the basic information of the patient, total medical expense, MI fund expense and OOP expense. NHSUS extracted data of medical expenditure based on the chief complaint for a visit to the hospital. Those hypertensive patients with medical cost for comorbidities during a visit were regarded as separate pieces of cost information in the database. Thus, the outpatient medical expenditure in our discussion covers only direct medical expenditure for hypertension and its complications, but not comorbidities.

### Statistical analysis

SPSS package (Version 12.0, SPSS Inc., Chicago, IL, USA) was used to perform statistical analyses. Continuous variables were compared using Student’s *t* test. A *p* value of less than 0.05 was considered statistically significant. Moreover, a linear regression analysis was done for total medical expense, MI fund expense and OOP expense to identify possible risk factors.

## Results

### Patient demographics

Among the 102,492 hypertensive outpatient visits, male patients account for 49,908 visits (48.69%) and female patients accounting for the rest 52.96%. After classifying these visits by payment method, patients in the FFS group account for 50,726 visits and patients in the GBPS group account for 51,766 visits. By patient’s unique identity number, the 102,492 visits can be attributed to 21,925 different patients. The average age of these patients are 61.05 years old, with those of male patients being 60.76 and female patients being 61.36. Detailed demographic features are shown in Table 1.

**Table 1:** Patient demographic characteristic

Characteristics	Total	FFS group	GBPS group
Number of visits	102,492	50,726	51,766
Number of patients	21,925	11,306	10,619
Average visits per capital	4.67	4.48	4.87
Age	61.05 ± 13.09	60.76 ± 13.04	61.36 ± 13.14
Sex ratio (M:F)	10,313:11,612	5226:6080	5087:5532

### Expenditure of health services

**Fig. 1:**
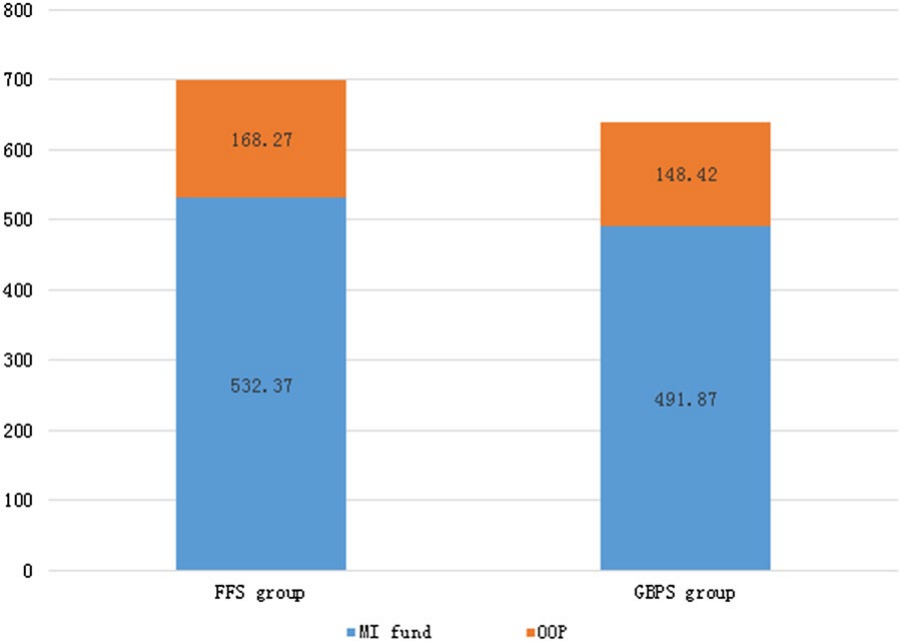
Changes of components of total medical expense due to payment method

For the expenditure of hypertension treatment per capital, average total medical expense, MI fund expense, OOP expense per capital and self-payment ratio were CNY671.40, CNY512.75, CNY158.65 and 22.26 respectively. A *t* test was done to compare the average medical expenditure between different groups of age, gender and payment method. Average total medical expense, MI fund expense, and OOP expense per capital were all highest for those above 65 years old, with those between 45 and 64 years old ranking the second and those between 18 and 44 years old the last. Self-payment ratios did not show significant difference between the age groups. As for the effect of gender, the above expenditure indexes are all higher for male patients than for female patients. As for the effect of payment method, the above expenditure indexes except for self-payment ratio are all significantly higher for those in the FFS group than those in the GBPS group. Detailed effects of age, gender and payment methods on medical expenditure are shown in Table 2. GBPS reduced the two components of total medical expense, meaning MI fund expense and OOP expense, to a similar extent, without significant change of the self-payment ratio. Changes of the two components of total medical expense due to payment method are shown in Fig. 1.

**Table 2:** Average expenditure per capital

Expenditure index	Total medical expense	MI fund expense	OOP expense	Self-payment ratio (%)
Total	671.40	512.75	158.65	22.26
Age				
18–44	487.49 ± 625.75	372.89 ± 475.27	114.60 ± 165.16	22.03% ± 12.12
45–64	678.54 ± 764.94***	517.77 ± 577.86***	160.77 ± 202.71***	22.26% ± 10.71
≥65	716.80 ± 805.29***	547.83 ± 609.62***	168.98 ± 210.90***	22.32% ± 10.15
Gender				
Male	694.94 ± 798.59	532.07 ± 605.79	162.87 ± 208.55	22.00% ± 11.01
Female	650.51 ± 741.60***	495.60 ± 559.26***	154.91 ± 197.19**	22.49% ± 10.36**
Method of payment				
FFS group	700.64 ± 760.66	532.37 ± 572.84	168.27 ± 204.76	22.32% ± 11.18
GBPS group	640.28 ± 777.08***	491.87 ± 590.67***	148.42 ± 199.88***	22.19% ± 10.10

Results are expressed as mean ± standard deviation. For comparison of average expenditure among different age groups, those patients between 18 and 44 years old were used as reference. For comparison of average expenditure between different gender groups, male patients were used as reference. For comparison of average expenditure between groups of different payment method, those patients in the FFS group were used as reference

*MI* medical insurance, *OOP* out-of-pocket

* p value < 0.05,** p value < 0.01,*** p value < 0.001

To rule out possible confounding effects from age and gender, total population was stratified by these factors and a *t* test was done in each layer of patients to further identify the influence of payment method. Results show that total medical expense, MI fund expense and OOP expense remain significantly lower for GBPS in all layers except those above 65 years old. Further examination of the data also shows that, the difference of average total medical expense, MI fund expense and OOP expense between FFS group and GBPS group all decrease as patients get older. Detailed comparison of payment method in each layer of patient is shown in Table 3.

**Table 3:** Average medical expenditure per capital stratified by age and gender

	Total medical expense			MI fund expense			OOP expense		
	FFS group	GBPS group	FFS-GBPS	FFS group	GBPS group	FFS-GBPS	FFS group	GBPS group	FFS-GBPS
Age									
18–44	541.23 ± 679.05	431.32 ± 559.47***	109.91	412.93 ± 514.74	331.03 ± 426.35***	81.90	128.29 ± 180.83	100.28 ± 145.72***	28.01
45–64	718.75 ± 764.08	632.91 ± 763.43***	85.84	545.49 ± 573.07	486.32 ± 581.70***	59.17	173.26 ± 209.02	146.59 ± 194.35***	26.67
≥65	723.49 ± 773.78	710.23 ± 835.12	22.26	550.16 ± 585.18	545.55 ± 632.76	4.61	173.34 ± 204.13	164.69 ± 217.29	8.65
Gender									
Male	734.59 ± 808.21	654.20 ± 786.60***	80.39	560.61 ± 609.88	502.75 ± 600.20***	57.86	173.78 ± 216.11	151.45 ± 199.86***	22.33
Female	671.45 ± 716.07	627.49 ± 768.08**	43.96	508.09 ± 537.83	481.86 ± 581.65*	26.23	163.36 ± 194.35	145.62 ± 199.88***	17.74

Results are expressed as mean ± standard deviation

*MI* medical insurance, *OOP* out-of-pocket

* p value < 0.05,** p value < 0.01,*** p value < 0.001

Moreover, generalized linear regression was conducted, using gender, age and method of payment as independent variables, and total medical expense, MI expense or OOP expense as dependent variable. The results conform to the above analysis that GBPS helps to reduce medical expenditure while male and older patients tend to cause higher expense. Further examination of the results show that, among these risk factors in the model, age is the single largest contributor for causing increasing medical expenditure. Detailed features of risk factors in the model are shown in Table 4.

**Table 4:** Parameter estimates for regression model of medical expenditures

	Total medical expense	MI fund expense	OOP expense
Age			
18–44	Referent	Referent	Referent
45–64	192.38***	146.14***	46.24***
≥65	230.95***	176.14***	54.82***
Female	−47.27***	−38.52***	−8.75**
GBPS group	−61.92***	−41.76***	−20.16***
R-squared	0.01	0.01	0.009

*MI* medical insurance, *OOP* out-of-pocket

* p value < 0.05,** p value < 0.01,*** p value < 0.001

## Discussion

### Demographic factors

Hypertension treatment expenditure is known to be associated with demographic factors such as age and gender. Our data confirms previous research that direct hypertension expenditure increases with age (Le et al. 2012). As hypertensive patients get older, severity of the disease and relative complications tend to increase, which can lead to concomitant rising expenditure. Moreover, the increasing trend of hypertension treatment cost applies to not only MI fund expenses, but also OOP expenses. Concerning the more vulnerable financial capacity of the elders, this result again emphasizes the need for inclining health insurance reimbursement for older hypertensive patients.

### Payment method

Results from our study show definite cost control effects of GBPS compared with traditional FFS, for total medical expense, MI fund expense and OOP expense. Except for those hypertensive patients above the age of 65 years old, the cost control effects of GBPS compared with OOP remain significant even after being stratified by age and gender. Qiu et al. also reported the cost control effects of GBPS for inpatient hospital expenditure from the analysis of 40 cerebral infarction patients in Tianjin (Qiu 2009). Total hospitalization cost per visit decreased by 8.7% from CNY10307.5 before the implementation of GBPS to CYN9411.0 after implementation. Along with total medical expense, drug use proportion and average length of hospital stay also decrease with GBPS. In this sense, by prospective payment, GBPS can be an effective method to curtail the fast rising medical expenditure and limit the incentive of health care providers to offer unnecessary service.

Moreover, GBPS shows robust cost control effects on the two components of total medical expense, meaning the MI fund expense and OOP expense. On one hand, MI fund expenses decrease significantly for GBPS compared with FFS, which can help minimize the risk of MI fund deficit and maximize the efficiency of insurance budget function. On the other hand, OOP expenses also decrease in the GBPS group to a similar extent of total medical expenses. Since the medical insurance system in China does not cover all expenses for patients, the affordability has always been a task of China’s health reform (Long et al. 2013). Increasing medical expenditure confers pressure not only for MI fund, but also for the financial vulnerability of low-income patients. Results from this study show enjoyable results that GBPS can also make hypertension treatment more affordable.

One interesting result from our study is that GBPS failed to decrease medical expenditure per capital for those above 65 years old. Concerning the fact that medical expenditure tends to increase with age, this result may imply the inability of GBPS to contain medical expenditure for those with an inherent high demand of medical service. The difference between GBPS and FFS for total medical expenses, MI fund expenses and OOP expenses also show a decreasing trends as patients get older. One possible explanation for this is that GBPS helps to restrain those unnecessary medical service, while the inherent demand of service due to severity of disease or relative complications can still amount to high level of expenditure. On the other hand, FFS has also set limitation on the maximum amount of reimbursement for a patient, and a negotiation between patients and health care providers may exist for reducing the volume of service as total expenses approaches the limitation. In this way FFS can also to some extent curtail the amount of service for those patients with high level of expenditure.

Though the cost control effects for inpatient expenses have been confirmed in initial studies (Song et al. 2014), the actual influence of GBPS on outpatient expenses is still not clear. Previous researches have predicted a shift of inpatient hospitalization to outpatient service by GBPS (Markovich 2012). However, our results prove a similar power of GBPS to limit unnecessary outpatient medical service in hypertension treatment, and this offers support for a wider implementation of GBPS.

Admittedly, GBPS may cause some adverse effects while reducing medical expenditure. For example, the average visits per capital for GBPS is a little bit more than that of FFS. The increasing times of visit may be a side effect of decreased medical service per visit, for a sense of health care providers to reduce expense for meeting the goal of the global budget may sometimes hypercorrect the former unnecessary expense by FFS. This hypercorrection, for example, reducing the amount of drugs prescribed per visit, can make the needs of some of the patients that indeed have a high demand of service unsatisfied, which will lead to increasing times of hospital visits. However, the average expense per visit for GBPS is smaller, which on the whole makes its total medical expense significantly lower.

There have been worries about quality of medical service and acceptance of health care providers since the introduction of GBPS. These concerns can be reasonable, for health care providers may reduce service quality to decline operating expenses, or shirk patients to other hospitals, so long as they can receive the same prepaid annual budget. Initial results from America show that GBPS can offer comparable quality of service and gain the acceptance of health care providers (Song et al. 2012, 2014). The key points in limiting the possible disadvantages of GBPS may lie in designing rational annual budget. A budget too high can decline the effects of cost control, while a budget too low may lead hospitals to reduce service quality. Moreover, there has been a recent trend for mixing prepaid payment method and FFS (Hsu 2014). With these in mind, HRSSB in Tianjin brings factors including service volume and capacity of a hospital into consideration when determining its annual budget, and reserves 6% of the annual budget for the year-end assessment, although further investigations are needed for better calculating the budget and evaluating the quality of service after implementation of GBPS.

A limitation of this study is that it is cross-sectional, which may neglect possible unbalance of base line characteristics between the GBPS group and FFS group. However, since it is a random selection of all patients to Tianjin’s primary hospital, this possibility of nonrandomization can be mitigated.

In conclusion, this study offers support for the cost control effects of GBPS, both for the MI fund expenses and OOP expenses, from the analysis of outpatient hypertension treatment data in Tianjin. The cost control effects of GBPS tend to decline for those with higher demand of medical service, and further studies are needed to analyze change of service quality after implementation of GBPS.
